# Precise dapagliflozin delivery by cardiac homing peptide functionalized mesoporous silica nanocarries for heart failure repair after myocardial infarction

**DOI:** 10.3389/fchem.2022.1013910

**Published:** 2022-11-04

**Authors:** Lijiao You, Qing Wang, Yuhui Ma, Yunfeng Li, Hui Ye, Lingli Xu, Ming Lei

**Affiliations:** Department of Critical Care Medicine, Seventh People’s Hospital Affiliated to Shanghai University of TCM, Shanghai, China

**Keywords:** myocardial infarction, dapagliflozin, nano-targeted drugs delivery system, heart failure repair, hypoxia-inducible factor 1-α (HIF-1α)

## Abstract

Myocardial infarction (MI) may cause irreversible damage or destroy to part of the heart muscle, affecting the heart’s ability and power to pump blood as efficiently as before, often resulting in heart failure (HF). Cardiomyocyte death and scar formation after MI may then trigger chronic neurohormonal activation and ventricular remodeling. We developed a biocompatible and mono-dispersed mesoporous silica nanoparticles (MSN) divergent porous channel for dapagliflozin (DAPA) loading. After surface modification of the cardiac-targeting peptides, the novel drug delivery system was successfully homed, and precisely released drugs for the hypoxic and weak acid damaged cardiomyocytes. Our biocompatible MSN- based nanocarriers for dapagliflozin delivery system could effective cardiac repair and regeneration *in vivo*, opening new opportunities for healing patients with ischemic heart disease in clinical.

## 1 Introduction

Myocardial infarction (MI) contributes to more than 40% of sudden cardiac deaths and represents the leading cause of morbidity and mortality worldwide (Sathisha et al., 2011; Nigam 2007; Anwar et al., 2016). In myocardial ischemia area, broadscale myocardium tissue are impaired with apoptotic/necrotic cardiomyocytes. Due to the low proliferation capability of myocardial cells, the damaged myocardium tissue is always unable to be effective regeneration and restoration (Zhao, et al., 2022). Meanwhile, complications of heart failure (HF) after MI hospitalization may cause cardiomyocyte apoptosis and scar formation, subsequently triggering chronic neurohormonal activation and ventricular remodeling (Dudas et al., 2011; Roger 2013; Desta et al., 2015; Jenča D et al., 2021). The high prevalence of HF complications after hospital discharge greatly reduces the quality of life of MI patients. It is estimated that approximately 13% of patients will experience HF complications 30 days post-discharge, with this number increasing to 20–30% in the first year after discharge (Hung et al., 2013; Sulo et al., 2016). Thus, HF is associated with three alarmingly high rates: high incidence rate, high mortality rate, and high rehospitalization rate. Scientists recently reported that the sodium-glucose cotransporter two inhibitor, dapagliflozin (DAPA) is a promising new drug for the treatment of HF. DAPA is approved by the Food and Drug Administration (FDA)—could reduce the risk of cardiovascular death and hospitalization for cardiac deterioration, increase survival rate and improve symptoms of HF in patients with decreasing ejection fraction (McMurray et al., 2019b, a). DAPA can effectively improve cardiac structure and function, weaken cardiac fibrosis and myocardial apoptosis, as well as inhibit inflammatory cytokines (Wang K et al., 2021). While the low selectivity, poor biodistribution and high systematic toxicity of DAPA greatly restrict its further clinical application (Wilczewska ZA, et al., 2012). Furthermore, treatment for MI is expensive and extensive, the prognosis is poor. Frequent episodes of HF and repeated emergency treatment in MI patients further increase the cost of treatment (Cowper et al., 2019). This situation led to the urgent construction of a novel drug delivery system (DDS) for HF repair. The novel DDS can be loaded with DAPA, which improves HF symptoms and increases patient survival. In addition, the novel DDS should be targeted precisely to improve drug utilization.

Novel drug delivery systems have been rapidly developed over the past few decades. Both native and synthetic nanomaterials have been extensively explored and proposed as the main components of DDS (Rodrigues et al., 2016; Swamy and Sinniah 2016; Mohanty et al., 2017). Recently, synthesized nanomaterials, including carbon materials (Bao R et al., 2017; Sun X et al., 2017), dopamine nanoparticles (Chakroborty et al., 2011; Das and Jana 2015), inorganic silica (Yang et al., 2010; Zhu et al., 2011), and polymeric assembly nanohybrids (Syed et al., 2012; Kerr et al., 2022), have been employed as nanocarriers for DDS. Porous silica has been recommended as an ideal DDS based on the biocompatibility of silica’s drug embedding ability in the mesoporous channel (Meng et al., 2010; Che et al., 2014; Chen K et al., 2020; Chen YJ et al., 2020). Furthermore, mesoporous silica nanospheres (MSNs) have been widely used for drug delivery because of their optimal surface area for drug loading, their facile synthesis and surface functionalization by anchoring targeting ligands, stimuli-responsive agents and sensing molecules (Sahoo et al., 2014; Sapino et al., 2015; Ugazio et al., 2016; Jafari et al., 2019). MSNs are considered biocompatible with the human body, as indicated by numerous investigations. The therapeutic dose of MSNs for DDS is well below the toxic level for humans, making MSN an excellent option for clinical applications.

Cardiac homing peptide (CHP) is a short peptide that preferentially binds to ischemic myocardium, and its sequence is CSTSMLKAC. CHP was first screened in a study of selective targeting of random peptides to ischemic tissue *in vivo* and was shown to be a safe peptide that did not cause any significant impairment of left ventricular systolic function. CHP has the potential to be used in the development of targeted therapy drugs for ischemic lesions of myocardial tissue (Kanki et al., 2011; Won et al., 2013; Vandergriff et al., 2018).

**FIGURE 1:**
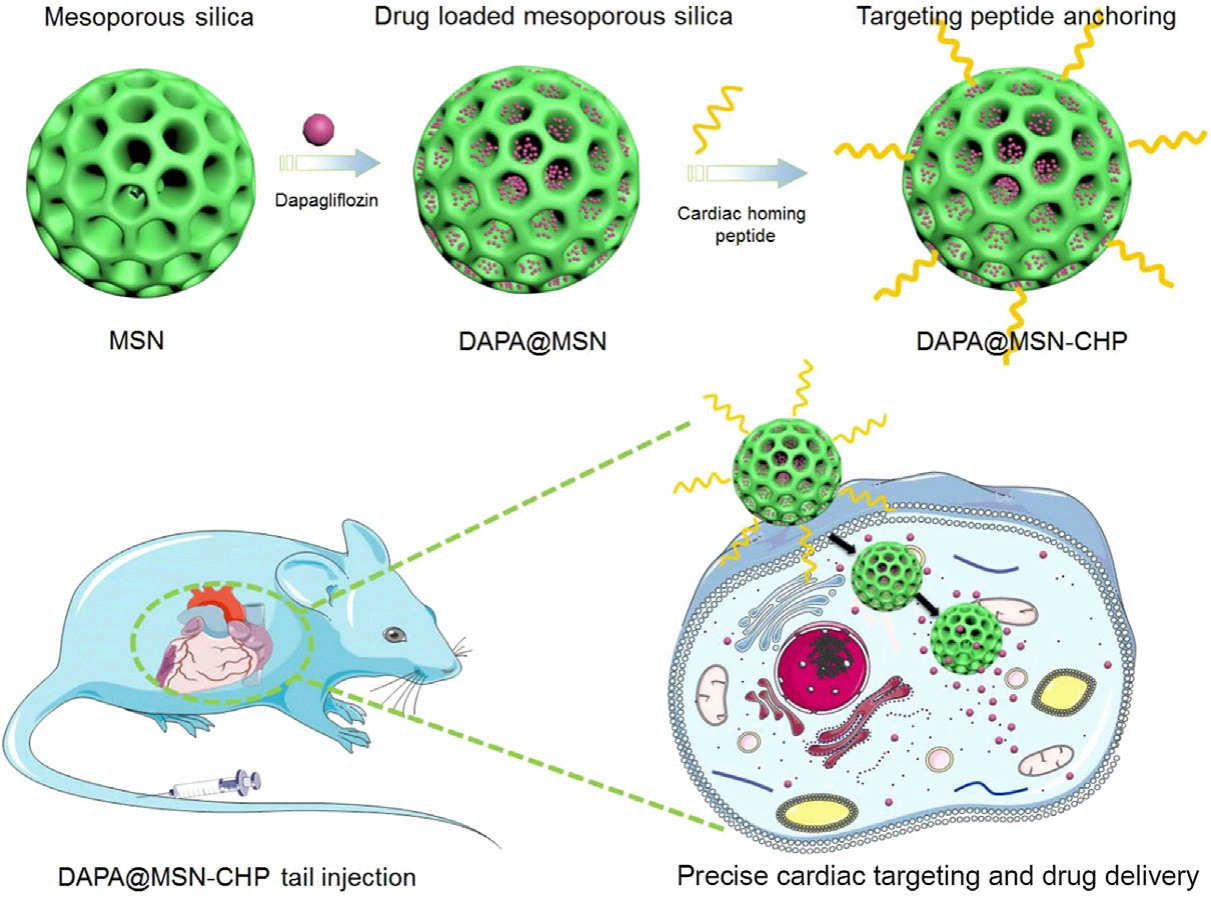
Dapagliflozin-loaded biocompatible mesoporous silica nanoparticles with surface functionalization of cardiac homing peptide fabrication for precise heart failure repair after myocardial infarction.

Here, we propose a conventional bi-phase approach for MSN synthesis for DAPA loading in heart failure repair. The hydrophobic and cation-π interaction properties of DAPA facilitate the efficient encapsulation of this drug in mesoporous channels (DAPA@MSN). Moreover, a CHP was functionalized on the surface of DAPA@MSN (DAPA@MSN-CHP). After intravenous injection of DAPA@MSN-CHP in a mouse model of HF after MI, this DDS efficiently and precisely accumulated in the HF site. DAPA-loaded MSNs have a slightly negative charge under normal physiological conditions but transform to a positive charge in the intracellular microenvironment of apoptotic cardiomyocytes because of the protonation effect under acidic conditions (Figure 1) (Wang et al., 2020). The results indicate that our DDS can effectively inhibit the apoptosis of cardiomyocytes, leading to viable myocardium preservation and cardiac function augmentation, laying a solid foundation for clinical HF repair.

## 2 Materials and characterization

Materials: Hexadecyltrimethylammonium bromide (CTAB), decahydronaphthalene, 1-octadecene (ODE) were purchased from Sigma-Aldrich. NaOH, cyclohexane and NH_4_NO_3_ were obtained from Shanghai Chemical Co., Ltd. Ammonia aqueous solution (28 wt%), tetraethyl orthosilicate (TEOS), (3-Aminopropyl) triethoxysilane (APTES), triethanolamine (TEA), decahydronaphthalene (98%) were purchased from Aladdin Industrial Inc. All chemicals were used as received without further purification.

Characterization: Transmission electron microscopy (TEM) measurements were carried out on a JEM 2100F microscope (Japan) operated at 200 kV. The samples were first dispersed in ethanol and then collected by using copper grids covered with carbon films for measurements. UV–vis–NIR absorption spectra were measured on a Shimadz spectrophotometer (UV-3150) (Japan) with wavelength range of 300 –1,200 nm, unless otherwise specified, all spectra were collected under identical experimental conditions. All animals underwent transthoracic echocardiography under anesthesia at 4 weeks after treatment using a VisualSonics Vevo 2,100 imaging system. The results were obtained by detecting Si content with inductively coupled plasma mass spectrometer in main organs.

### 2.1 Synthesis of mesoporous silica nanoparticles loading with DAPA and targeting peptides modification

#### 2.1.1 Mesoporous silica fabrication

The mesoporous silica nanoparticles were synthesized as the procedure reported previously. Briefly, 16 ml of (25 wt%) CTAB solution and 90 mg of triethanolamine were added to 20 ml of water and stirred gently at 60°C for 1 h in flask, then 1.5 ml of TEOS and 1 ml of cyclohexane was added to the solution and kept at 60°C with magnetic stirring for 24 h. The products were collected by centrifuging and washed by water and ethanol for several times, and then were dispersed in 30 ml of acetone and refluxed for 8 h to remove CTAB templates. The final products were washed with ethanol and dried in vacuum at 45°C for 8 h.

#### 2.1.2 Loading of dapagliflozin (DAPA)

DAPA was dissolved in DMSO (2.0 ml). Mesoporous silica nanoparticles (5.0 mg) were added to the solution and the suspension was stirred at room temperature for 48 h. The DAPA molecules could be adsorbed in the mesopore channels. The as-prepared DAPA-loaded mesoporous silica nanoparticles were collected by centrifugation. The amount of the adsorbed guests was determined from the difference between the initial amounts of DAPA by measuring the UV absorbance from the supernatant liquid and quantified from a standard curve.

#### 2.1.3 Cardiac homing peptides modification

To functionalization of DAPA@MSN, 3.0 g of silica was placed in a 100-ml round bottom flask with 50 ml of dry toluene and 2.4 g of 3-aminopropyltriethoxysilane (APTES). The mixture was stirred and refluxed at 110°C for 48 h under the protection of nitrogen. The white 3-aminopropyl functional silica washed with toluene, *n*-hexane and dichloromethane, respectively, and dried at 25°C for 12 h.

#### 2.1.4 DAPA@MSN-CHP fabrication

First, 20 mg CHP, 10 mg EDC and 8 mg NHS were dissolved in 20 ml anhydrous DMF, and incubated at room temperature overnight to activate the carboxyl groups of FA. Then, DAPA@MSN with NH_2_ modification (20 mg) was added. The mixed solution was allowed to react for 12 h, and the DAPA@MSN-CHP were obtained by centrifugation at 12,000 rpm for 20 min, and then washed with methanol three times.

### 2.2 *In vitro* cellular targeting and cell viability of DAPA@MSN-CHP

#### 2.2.1 Cell viability

All experiments were carried in 96-well plates. Cytotoxicity of MSN-CHP was tested *via* CCK-8 assay. Briefly, the primary cardiomyocytes isolated through enzymatic digestion were seeded into plate at 5×10^3^/well in 100 μL of DMEM/Low glucose (10% FBS, 100 units/mL of penicillin and 100 μg/ml of streptomycin), and incubated for 24 h. Then, 10 μL CCK-8 solution was added followed by12 h incubation. After 12 h incubation, the absorbance of each well at wavelength of 450 nm was measured using a microplate reader. Data were presented as mean ± SD (*n* = 3).

#### 2.2.2 CLSM images of cellular uptake

The cellular uptake of DAPA@MSN-CHP was observed and imaged by confocal laser scanning microscopy (CLSM). Briefly, primary cardiomyocytes were seeded into 6-well plates at a density of 1×10^5^/well with 1 ml of DMEM/Low glucose (10% FBS, 100 units/mL of penicillin and 100 μg/ml of streptomycin) media and incubated for 24 h. After the treatment with DAPA@MSN-CHP, DAPA@MSN at a final concentration of 100 μg/ml, the cardiomyocytes were incubated for 12 h. Then, the cardiomyocytes were washed with PBS for three times and DAPI (1 μg/ml in PBS) was used to stain nuclei for 30 min prior to being observed under CLSM.

#### 2.2.3 Western-blot analysis

Cells were collected after 12 h incubation with PBS, DAPA, DAPA@MSN, and DAPA@MSN-CHP. Samples containing equal amounts of protein were electrophoresed using 10% SDS-PAGE, transferred onto polyvinylidene fluoride membranes and then incubated with specific primary antibodies. The blots were reacted with horseradish peroxidase-conjugated secondary antibodies and were detected using the enhanced chemiluminescence system (Santa Cruz Biotechnology, Inc.). The density of the band was quantified by densitometry and exposed to X-ray film (Eastman-Kodak, Rochester, NY, United States) using GAPDH levels as a control.

### 2.3 *In vivo* HF repair capability of DAPA@MSN-CHP

#### 2.3.1 *In vivo* HF repair

Animal experiments were approved by the Animal Research Ethics Committee of Shanghai Seventh People’s Hospital. Animal experiments were performed according to the animal use and care regulation and the animal management rules of the ministry of health of the People’s Republic of China. C57BL/6 mice (8–10 weeks old) were bought from Shanghai Institute of Family Planning Science Laboratory Animal management Department. (Shanghai, China). All efforts were made to minimize animal suffering. The myocardial ischemia model was constructed based on previously reported works. A total of 70 male 8- to 10-week-old C57 mice were used for this work (Luo L et al., 2016; Snider JC et al., 2021). For the myocardial ischemia model construction, all mice were received permanent coronary artery ligation. Mice were anesthetized with 160 mg/kg barbital sodium *via* intraperitoneal injection. Typically, before preparing the surgical model, ventilation system after endotracheal intubation is recommend to use when coronary artery ligation can be successfully and routinely performing the within 3 min. Subsequently, a 1.2 cm of small skin cut was conducted over the left chest according previously reported. After the dissection, both pectoral major and minor muscle retracted, then the fourth intercostal space can be successfully observed. Simultaneously, in order to open both the pleural membrane and pericardium, a mosquito clamp was used to cut a small hole at this intercostal space. When the clamp was slightly opened, partial heart section was gently squeezed out through this hole. The coronary artery ligation was carefully located, sutured, and ligated (from its origin ≈3 mm) by a commonly used 6–0 silk suture. The surgical ligation was confirmed successfully when the anterior wall from left ventricular changed to pale. Immediately, the heart was put back to form the intrathoracic space followed by air manual evacuation and muscle and the skin closure through the previously used purse-string suture. During the recovery durations, the mice were allowed to breathe and carefully monitored, which was usually completed within 3–5 min. The artificial respiratory aid was not allowed during the recovery period. For the DAPA@MSN-CHP induced therapy, the C57BL/6 mice with myocardial ischemia were divided into four groups including the MI, DAPA, DAPA@MSN, and DAPA@MSN-CHP. Meanwhile, health mice were set as control group. Each group contained 4 mice. Each group contained 4 mice. The MI, MI + DAPA, MI + DAPA@MSN, and MI + DAPA@MSN-CHP groups were treated with 100 μL of PBS, DAPA, DAPA@MSN, and DAPA@MSN-CHP solutions *via* tail injection, respectively one time every day for 3 days. Meanwhile, the control groups were also received tail vein injection of PBS one time every day for 3 days.

#### 2.3.2 Cardiac function assessment

All animals underwent transthoracic echocardiography under anesthesia at 4 weeks after treatment using a VisualSonics Vevo 2,100 imaging system. During ultrasound process, mice were anesthetized with 3% isofluorane *via* a R500-Comapct Small Animal Anesthesia Machine (Shenzhen, China). Hearts were imaged 2D in long-axis views at the level of the greatest left ventricular diameter. Estimation of the function and fractional shortening were determined by measurement from views taken from the infarcted area. All measurements were done in random order, with the surgeon and echocardiographer being blind to the treatment groups.

#### 2.3.3 Histopathological evaluation

Hearts were harvested and cut into 10 μm-thick tissue sections. H&E and Masson’s trichrome staining of normal, MI, DAPA, DAPA@MSN, and DAPA@MSN-CHP was performed. Image analysis related to viable myocardium and scar size was performed using NIH ImageJ software.

## 3 Results and discussion

### 3.1 DAPA@MSN-CHP fabrication and characterization

**FIGURE 2:**
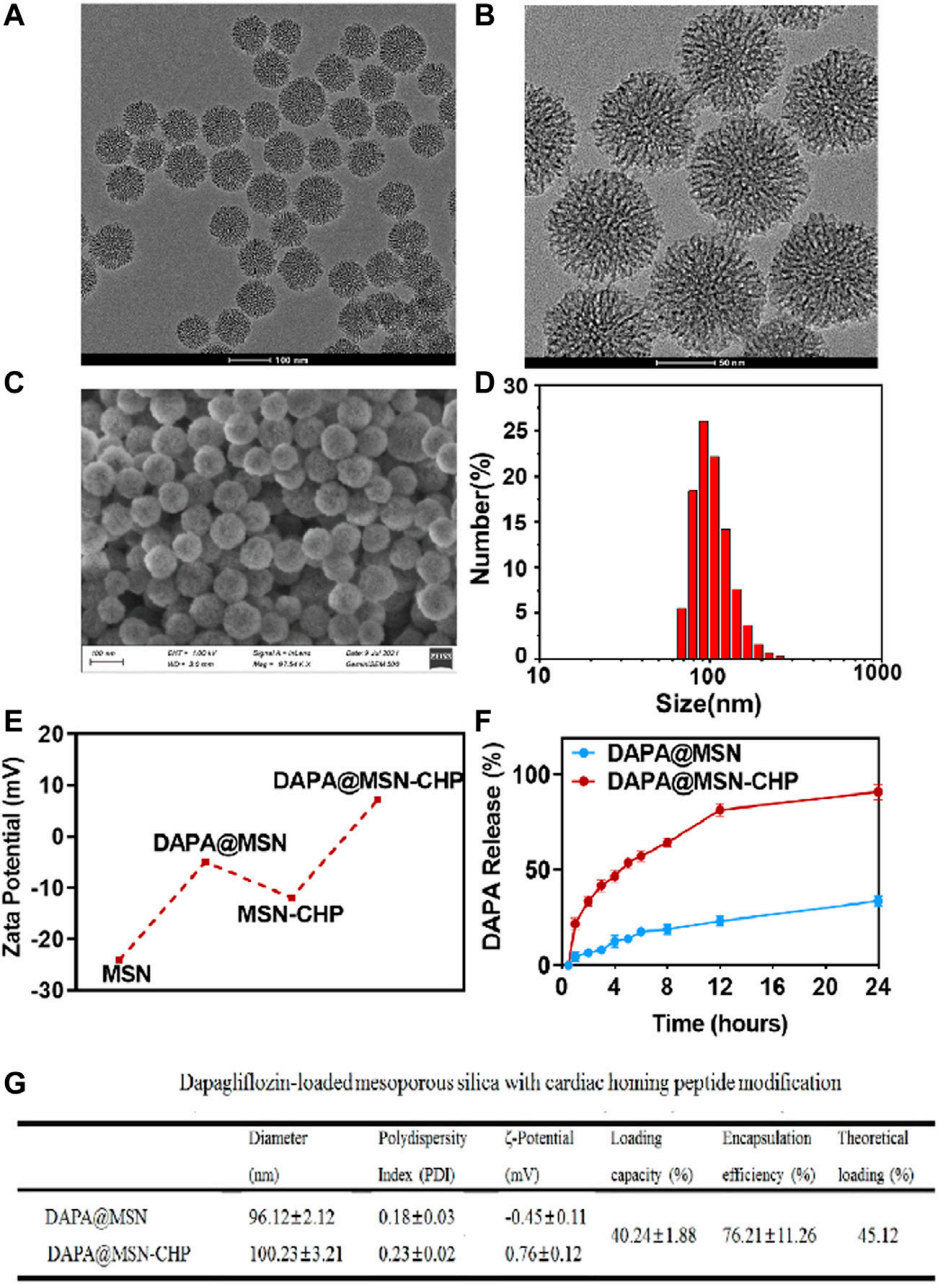
TEM image **(A)**, magnification TEM image **(B)** and SEM image of the bi-phase prepared MSN **(C)**. DLS result of size distribution of MSN **(D)**. Zeta-potential data of MSN, DAPA@MSN, MSN-CHP and DAPA@MSN-CHP **(E)**. DAPA release curve of DAPA@MSN-CHP under different pH values **(F)**. The corresponding physical and chemical parameters of the DDS **(G)**.

MSNs were fabricated using a novel biphase approach in a cyclohexane and water stratification system (Shen et al., 2014). The biphase stratification approach enables hydrolysis precursors in the interface. It conveniently regulates the nanoparticle assembly in the biphase interface by adding or changing other reactants in two different phases without affecting interfacial tension. The hydrophobic upper layer consisted of 25% tetraethyl orthosilicate (TEOS) solution in cyclohexane. The lower hydrophilic layer was a pure water solution mixed with cationic cetyl trimethylammonium cetyl bromide (CTAB) as the template and surfactant and 25% organic weak base triethanolamine (TEA) as a reducing agent. The dendritic hierarchical mesostructure with monodispersion was obtained *via* continuous interfacial growth in a facile one-pot strategy for 48 h (Figure 2A). A divergent mesoporous channel could be observed in the magnification transmission electron microscope (TEM) image (Figure 2B). The MSN pore size can be altered by changing the type of hydrophobic solvent in the upper layer.

The surface morphology of the MSN was also investigated using a scanning electron microscope (Figure 2C). The properties of the mesoporous channels and their uniform size were detected. The size distribution of our MSNs were also estimated by dynamic light scattering (DLS). Results showed that the drug delivery carriers had a narrow unimodal size distribution with a ∼96.2 nm peak, suggesting superior dispersity and uniform practical diameter (Figure 2D). Subsequently, the MSN surface was modified with the amino group in the ethanol solution through aminopropyltriethoxysilane hydrolysis. In the following step, DAPA was encapsulated in the mesoporous channels. Then, CHP could be anchored on the silica surface by EDC/NHS reaction between the MSN amino group and the carboxyl group of the targeting peptides.

Zeta potential of MSN presents ∼ -25 mV, contributing to the massive silicon hydroxyl group on the silica surface (Figure 2E). Interestingly, compared with free MSN, DAPA@MSN exhibits a weaker negative charge, mainly due to the positive charge of the amino group modification. DAPA@MSN-CHP has a positive charge in the aqueous solution, mainly because of the amino group consumption after the condensation reaction. The dominant positive charge of our DDS may originate from CHP, which facilitates the endocytosis of the DDS. The corresponding physical and chemical parameters of the DDS are listed in Figure 2G, showing all the sizes fluctuate minimally after drug loading or CHP modification. Loading efficiency meets theoretical efficiency estimations, further demonstrating that MSNs are the ideal nanocarriers for drug delivery. Li ZX et al. reported that small nanoparticles (< 200–300 nm) are normally taken up by cells *via* the endocytic pathway. Particle size, particle shape, and particle surface charge have a certain impact on the endocytic uptake of cells. Generally, particles with small particle size, rough surface and positively charged surface are more likely to be phagocytosed by cells (Li et al., 2012). The characterization results of the above materials suggest that DAPA@MSN-CHP may have good biocompatibility thanks to its small nanoparticle size, rough mesoporous surface, and positive charge characteristics.

MI leads to changes in the microenvironment at the site of cardiac injury. Constructing acidic pH-responsive DDS based on the weakly acidic microenvironmental characteristics of lesions can improve the value of drug utilization (Li et al., 2021). Finally, we investigated the drug release ability of DAPA@MSN-CHP under different pH conditions. Compared with the normal pH value of ∼7.2, 92.1% of DAPA can be released after 24 h incubation in biological buffers with pH values of ∼5.5 (Figure 2F). These results suggest that DAPA can continuously release under the weak acid in the HF site after MI which is beneficial to improve the utilization value of drugs.

### 3.2 *In vitro* investigation of DAPA@MSN-CHP

**FIGURE 3:**
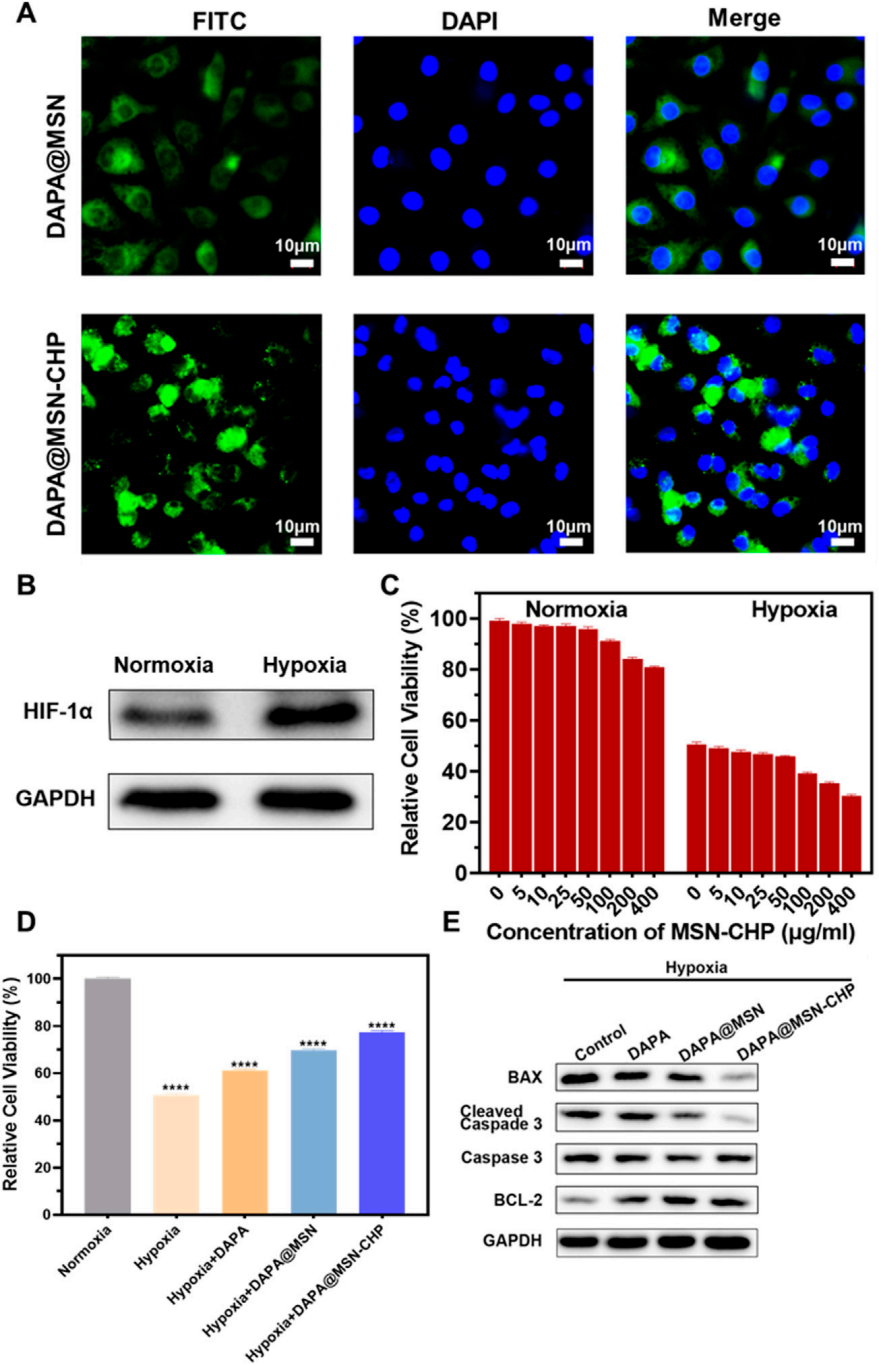
CLSM images of DAPA@MSN-CHP and DAPA@MSN with 12 h incubation **(A)**. Western blot result of HIF-1α expression in HF tissues and normal tissue **(B)**. Cell viabilities of cardiomyocytes toward MSN-CHP under hypoxia and normoxia conditions **(C)**. Cell viabilities of cardiomyocytes after different treatments in hypoxic conditions. Pure normoxia condition was set as the control group **(D)**. Bax, cleaved Caspase-3, Caspase-3 and BCL-2 expressions of cardiomyocytes after different treatments under hypoxia conditions **(E)**. All error bars represent mean ± SD (*n* = 4). Data analysis was performed using one-way ANOVA, ^**^ *p* < 0 01, ^***^ *p* < 0 001.

Following the excellent performance of DAPA@MSN-CHP, we then estimated the intracellular localization of DDS in cardiomyocytes. DAPA@MSN and DAPA@MSN-CHP were co-loaded with Fluorescein isothiocyanate (FITC) for intuitive observation under a Confocal Laser Scanning Microscope (CLSM). They were then added to two groups of cardiomyocytes for 12 h before CLSM observation. FITC signals at 550 nm were collected under 488 nm excitation. As shown in Figure 3A, DAPA@MSN-CHP presents stronger fluorescent spots than DAPA@MSN with no targeted protein modification. The fluorescent spots of DAPA@MSN-CHP were mainly localized in the cytoplasm. At present, the target receptor of CHP and the mechanism of its specific targeting to ischemic tissue are not yet clear, but CHP has been proved to have good targeting delivery ability in a number of studies on ischemic myocardial targeted substance delivery systems. In a gene delivery study, Young-Wook Won et al. used CHP as a guide to achieve increased gene expression in H9C2 cells under hypoxic conditions (Won et al., 2013). Another study reported CHP as a targeting peptide to increase the uptake of peptide-labeled exosomes by oxidatively damaged H9C2 cells (Vandergriff et al., 2018). Our findings also indicate the precise binding ability of CHP to the receptor on the membrane of cardiomyocytes.

Previous reports indicate that hypoxia also affects most sites of heart diseases such as HF, cardiac arrest and heart attack (Jianqiang et al., 2015; Amofa et al., 2017; Hesse et al., 2018). The hypoxic conditions may originate from inadequate blood oxygen concentration delivery following vessel impairment in the damaged tissues. Furthermore, Hypoxia-inducible factor 1-α (HIF-1α) promotes the adaptation to hypoxia-related stress through increasing oxygen delivery and decreasing oxygen consumption (Anand et al., 2007; Kumar et al., 2020; Nguyen et al., 2021). Accordingly, H9C2 cells were treated with hypoxia, and than the expression level of HIF-1α was significantly increased (Figure 3B), indicating the successful establishment of myocardial hypoxia model. Based on this, we used the cardiomyocyte hypoxia model to study the effect of DAPA@MSN-CHP *in vitro*.

We then explored cell viability of cardiomyocytes under conditions of both normoxia and hypoxia based on our MSN-CHP. Figure 3C shows negligible cytotoxicity with the DDS concentration as high as 400 μg/ml in both normoxia and hypoxia, compared with a control group. These findings further demonstrate the superior biocompatibility of the MSN. Cell viability of our DDS was also studied under various conditions. Our DDS promoted significantly higher cell activity in hypoxia (∼77%). In contrast, only ∼51%, 61%, 70% of hypoxic cells survived in the solvent, DAPA, and DAPA@MSN incubation conditions, respectively (Figure 3D). This result derives from the efficient and precise release of our DDS. DAPA successfully transported and gradually released the drug in the weak acid intracellular microenvironment of HF cells in DAPA@MSN-CHP. Simultaneously, western-blot results demonstrated that our DDS improved the expression of cell survival-promoting factor (BCL-2) while reducing the expression of apoptotic factors Caspase-3 and BAX (Figure 3E). DAPA has been found to be effective in reducing cardiotoxicity and inhibiting apoptosis *in vitro* (Chang et al. 2022). Our results suggesting that the precise targeted delivery of DDS enhanced the intervention effect of DAPA on hypoxia-induced cardiomyocyte apoptosis.

### 3.3 *In vivo* HF repair capability of DAPA@MSN-CHP

**FIGURE 4:**
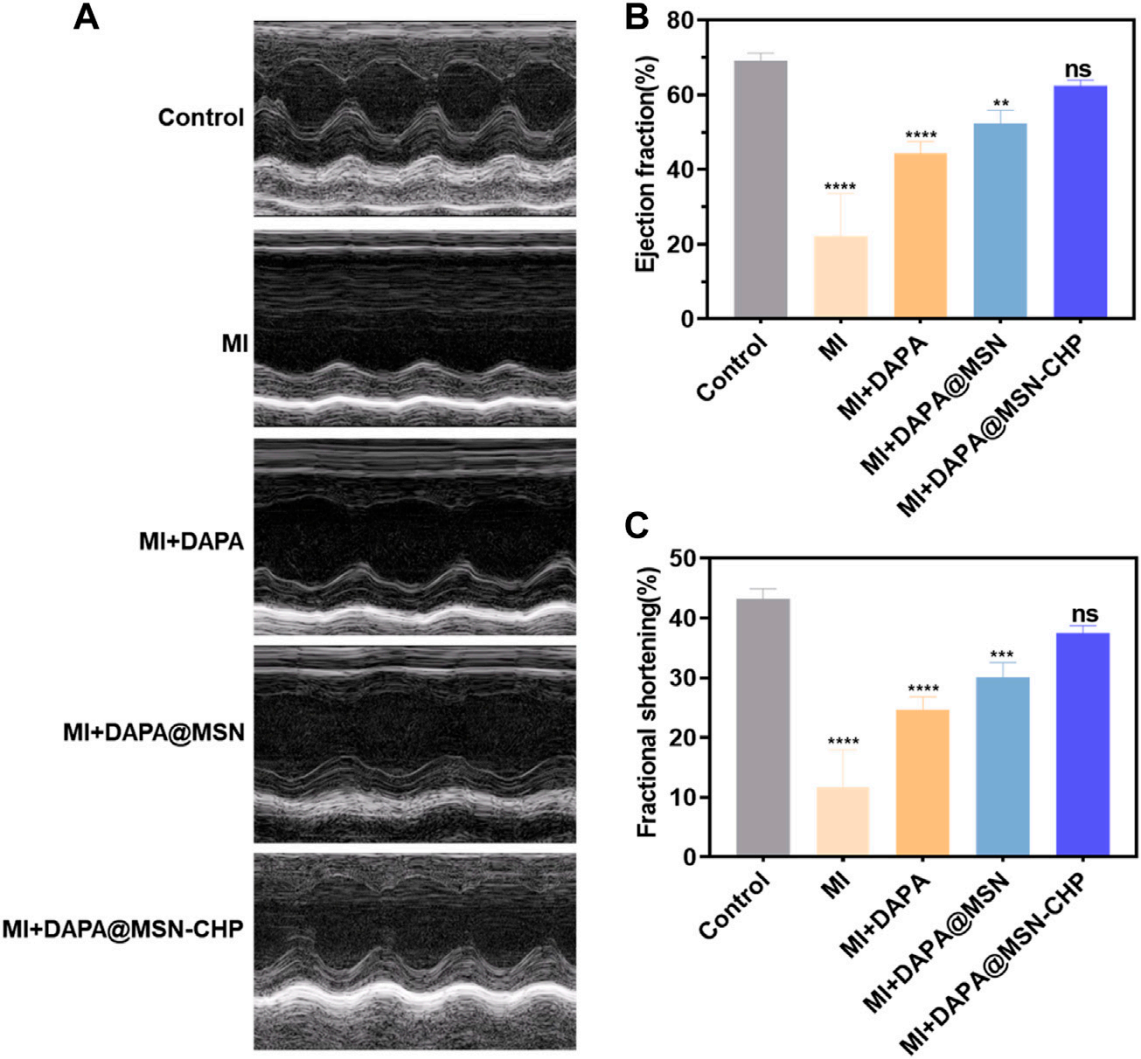
M-mode echocardiogram representative images of MI, MI + DAPA, MI + DAPA@MSN and MI + DAPA@MSN-CHP treatments. The healthy mice were set as controls **(A)**. Estimation of the function of MI hearts by ejection fraction **(B)** and fractional shortening **(C)** after MI, MI + DAPA, MI + DAPA@MSN and MI + DAPA@MSN-CHP treatments. The healthy mice were set as controls. All error bars represent mean ± SD (*n* = 4). Data analysis was performed *via* one-way ANOVA, ^**^ *p* < 01, ^***^ *p* < 001.

Since the targeting mechanism of CHP is not yet clear, the *in vitro* results are insufficient to demonstrate the targeting ability of DAPA@MSN-CHP. Therefore, we investigated HF repair efficiency using an MI mice model. The MI model was constructed with a temporary ligation of the left anterior descending coronary artery for 0.5 h. These mice were divided into four groups: pure MI, free DAPA, DAPA@MSN and DAPA@MSN-CHP groups, with normal mice as the control group (Figure 4A). Body distribution of DAPA@MSN-CHP results shows that the novel DDS had the highest cardiac-targeting efficiency compared with DAPA and DAPA@MSN (Supplementary Figure S1). Studies have reported changes in protein expression and protein distribution in cardiomyocytes under ischemia and hypoxia conditions (Liu et al., 2014), suggesting that the unknown receptor protein of CHP may increase its binding to CHP through these changes to play a specific targeting role. In the study of Young-Wook Won et al. and Adam Vandergriff et al., CHP as a component of a drug delivery system was also reported to play an active role in targeting MI tissue *in vivo* (Won et al., 2013; Vandergriff et al., 2018).

Studies have shown that DAPA reduces heart failure exacerbations and improves symptoms in patients with heart failure and reduced ejection fraction (Kosiborod et al., 2020). After 1 month, the therapeutic efficiency of DDS was evaluated by detailed echocardiography analysis. The heart functions were carefully estimated by conventional M-mode echocardiography. Compared with the healthy group, the interventricular septum of the MI group without any administration became weak as septal amplitude decreased, proving the persistent and serious avascular necrosis in myocardium tissue. Compared with the no treatment condition, the morphology of the myocardial layer gradually improved in the echocardiogram image of the DAPA and DAPA@MSN groups. DAPA@MSN-CHP treatment significantly restored hearts, as shown by comparison with healthy hearts.

The conventional indicators of heart function were subsequently evaluated. The ejection fraction values of the left ventricle in normal, MI, DAPA, DAPA@MSN and DAPA@MSN-CHP groups were 69.2%, 22.3%, 44.6%, 50.8% and 63.1%, respectively (Figure 4B). Similar trend was also detected in the ultrasound photographs of whole heart that MI + DAPA@MSN-CHP displayed most effective MI repair capability with highest ejection fraction values (Supplementary Figure S2). The fractional shortening values of the left ventricle in healthy, MI, DAPA, DAPA@MSN and DAPA@MSN-CHP groups were 42.3%, 12.3%, 24.8%, 29.7% and 37.6%, respectively (Figure 4C). In contrast with the MI, DAPA, and DAPA@MSN groups, the DAPA@MSN-CHP treatment resulted in optimal performance in restoring both ejection fraction and fractional shortening. There was no significant difference between the normal group and the DAPA@MSN-CHP groups, demonstrating the superior repair capability of the DDS.

### 3.4 Histopathological evaluation of DAPA@MSN-CHP for HF repair

**FIGURE 5:**
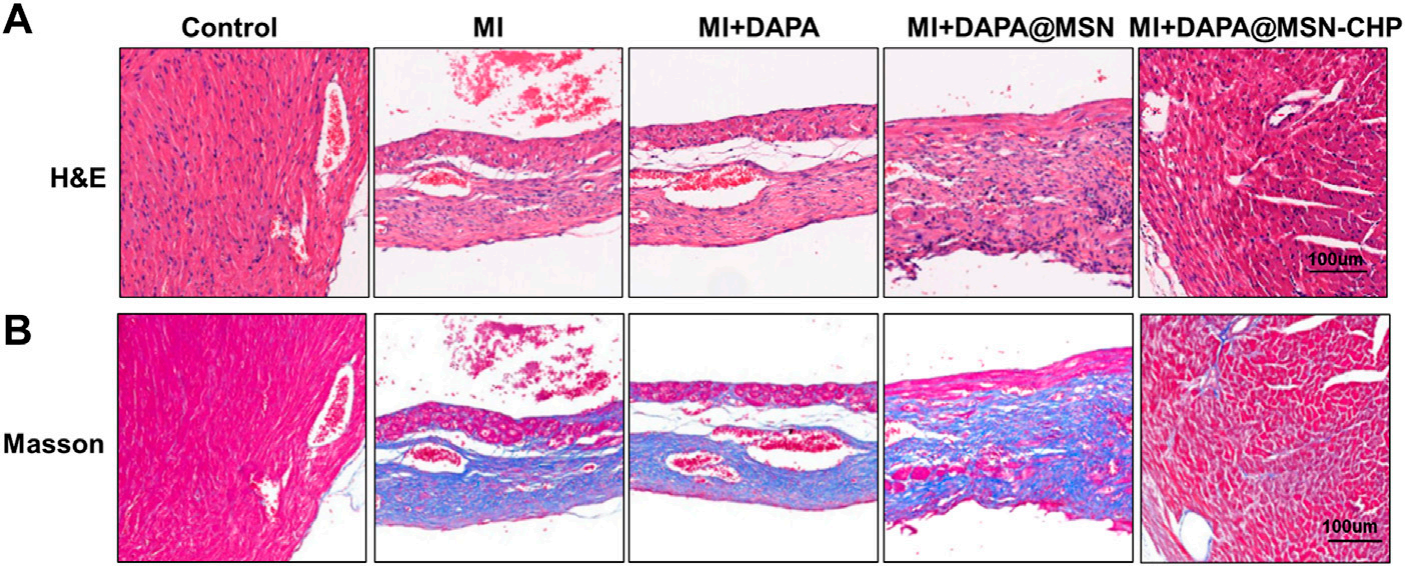
Representative H & E images **(A)** Masson’s trichrome stain images **(B)** of cardiac sections after MI, MI + DAPA, MI + DAPA@MSN and MI + DAPA@MSN-CHP treatments. The healthy mice were set as controls.

In the process of heart failure, pathological changes in cardiac tissue occur, mainly manifested as myocardial remodeling and inflammatory infiltration (de Boer et al., 2019). After various administrations, heart sections were stained with hematoxylin and eosin (H & E). The DAPA@MSN-CHP administration group was similar to the healthy mice and demonstrated a normal histoarchitecture with intact myocardial membranes, similar oval nuclei and regular cross striations (Figure 5A). In contrast, other groups showed histological alterations with myocardial separation and few scattered inflammatory cells. These atypical pathological phenomena were mainly caused by inflammatory leukocytes and wavy fibers. Masson’s trichrome staining of heart tissue was also used to validate the associated collagen deposits and fibrotic transformation associated with MI. Pure MI, DAPA, and DAPA@MSN showed thick and dense interstitial collagen fibrils (blue) related to necrotic myocytes. DAPA@MSN-CHP and control groups exhibited normal heart histoarchitecture with slight interstitial collagen fibrils stained with blue (Figure 5B). All histopathological examination results demonstrated the superior therapeutic efficiency of MSN-based DDS to repair cardiac function following MI.

### 3.5 Biocompatibility evaluation *in vivo*

**FIGURE 6:**
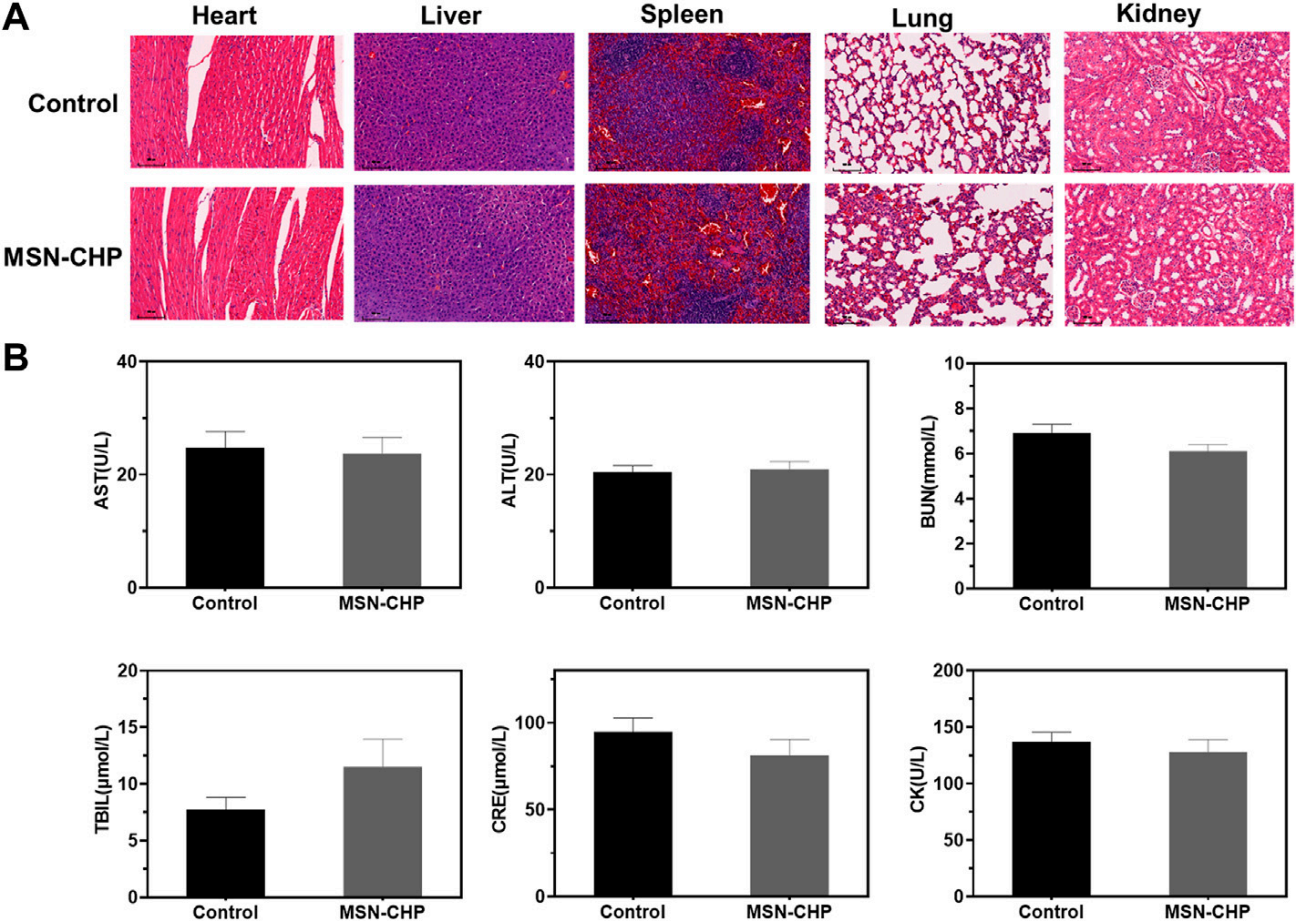
H&E-stained photographs of major organs after 3 days treatment of PBS and MSN-CHP **(A)**. Blood biochemistry results from PBS and MSN-CHP treated mice, respectively **(B)**.

Finally, we herein evaluate the systematic toxicity of our MSN-based DDS *via* H&E tissue staining analysis and blood biochemistry assesement. The tissue of main orans including heart, liver, lung, spleen and kidney were dissected after the tail vein injection of MSN-CHP and then they were stained by H&E. As shown in Figure 6A, there was ignorable tissue damage in MI, DDS treated group, as compared with the control group (normal mice), testifying the superior biocompatibility of our MSN-based nanocarriers. For the blood biochemistry analysis, as displayed as liver functional makers including ALT (alanine aminotransferase), AST (asparatate aminotransferase), ALP (alkaline phosphatase), TBIL (total bilirubin), BUN (Blood urea nitrogen) and CRE (creatinine), no any variations of hepatic toxicity were found after MSN-CHP administration (Figure 6B). As indicator for heart muscle damage, CK (creatine kinase) value in the blood of MSN based DDS treated mice was also maintained at normal level (Figure 6B). The above results provide a validation that MSN-based nanocarriers bore no remarkable side effect *in vivo*.

## 4 Conclusion

We developed a straightforward approach to fabricate biocompatible and monodispersed MSNs of negligible toxicity. Their large surface area facilitated the DAPA encapsulation in the mesoporous channel, and the nanocarrier surface was then functionalized for cardiac-targeting peptides. The DDS was highly efficient in MI region in HF, and the DAPA could be precisely released in the hypoxic, apoptotic and weak acid intracellular environment. DAPA@MSN-CHP demonstrated optimal therapeutic efficiency toward MI model mice, restoring the MI hearts and making them comparable with those of the healthy group. MSN-based DDS provides a possible application of precise and effective repair of MI in clinical.

## Data Availability

The original contributions presented in the study are included in the article/Supplementary Material, further inquiries can be directed to the corresponding authors.
