# Supplementary material for: Precise dapagliflozin delivery by cardiac homing peptide functionalized mesoporous silica nanocarries for heart failure repair after myocardial infarction
Source: Front Chem. 2022 Nov 4;10:1013910. doi: 10.3389/fchem.2022.1013910 (PMC9671955; doi:10.3389/fchem.2022.1013910)
Supplement: Supplementary file 1 [file DataSheet1.docx]

Precise Dapagliflozin Delivery by Cardiac Homing Peptide Functionalized Biocompatible Mesoporous Silica Nanoparticles Nanocarries with Dapagliflozin Delivery system fabrication for heart failure repair after myocardial infarction

## Supplementary Figures


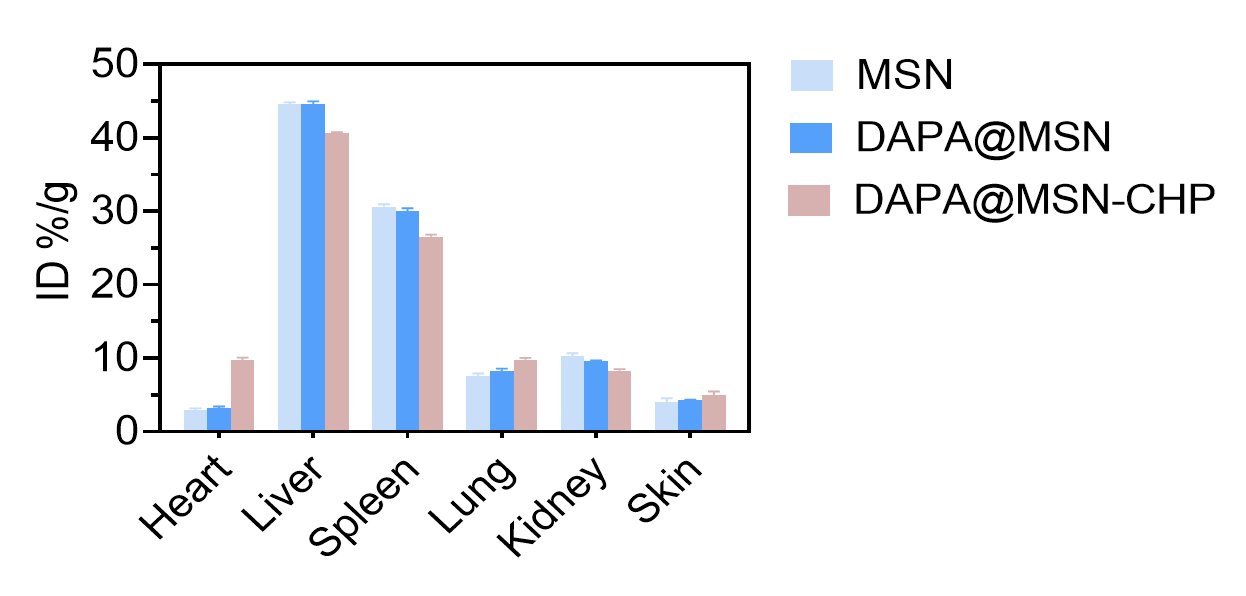


**Supplementary Figure 1.** Body distribution of MSN, DAPA@MSN, dapa@MSN-CHP treated MI mice after 12 h. The results were obtained by detecting Si content with inductively coupled plasma mass spectrometer in main organs.


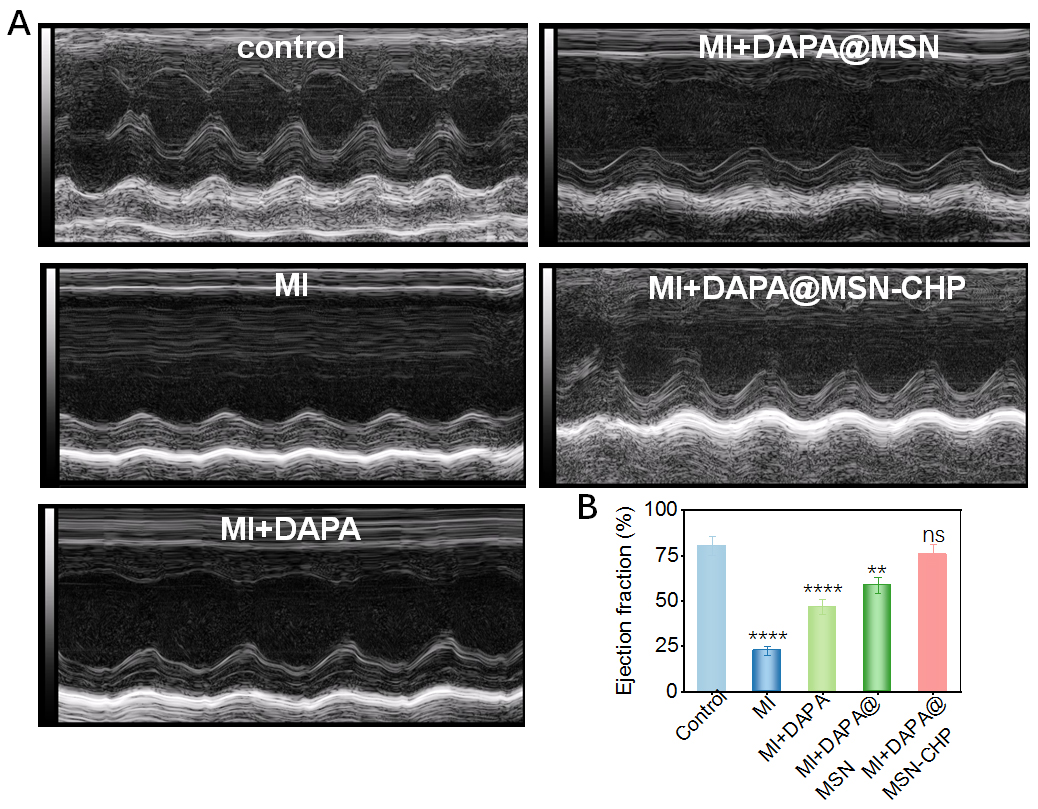


**Supplementary Figure 2.** M-mode echocardiogram representative images of whole heart after MI, MI+DAPA, MI+DAPA@MSN and MI+DAPA@MSN-CHP treatments. The healthy mice were set as controls (A). Estimation of the function of MI hearts by ejection fraction (B) after MI, MI+DAPA, MI+DAPA@MSN and MI+DAPA@MSN-CHP treatments.
